# Cognitive Behavioural Therapy Versus Psychodynamic Therapy for Medically Unexplained Symptoms: A Retrospective Study of Healthcare Utilisation and Cost Analysis

**DOI:** 10.1192/bjo.2024.218

**Published:** 2024-08-01

**Authors:** Rory Naylor

**Affiliations:** University of Oxford, Oxford, United Kingdom

## Abstract

**Aims:**

Patients experiencing Medically Unexplained Symptoms (MUS) are some of the costliest in both primary and secondary care. Psychotherapy is one of the most efficacious ways of treating them although the most superior modality is unclear. Cognitive Behavioural Therapy (CBT) has the greatest evidence base, but a growing number of studies have investigated the role of Psychodynamic Psychotherapy (PPT). This is the first study to compare the two modalities concerning their impact on healthcare utilisation and cost to the NHS.

**Methods:**

Patients referred to the Oxford Community Psychological Medicine Service in 2021 and who went on to complete a course of psychotherapy for MUS were included. 78 patients were referred, 66 patients were assessed, 16 patients began treatment and 9 patients completed treatment. 4 received CBT and 5 received PPT based on a ‘best fit’ assessment. Their healthcare utilisation (GP appointments, health investigations, A&E attendances, inpatient admissions and outpatient appointments) was assessed during the 6 months prior to their initial assessment and compared with the 6 months after therapy had ended using data from ‘Health Information Exchange’.

**Results:**

Overall, psychotherapy reduced primary care use but our data was insufficiently powered for this to be statistically significant. There was a significant reduction in outpatient appointments after psychotherapy, mostly representing mental health consults.

Significant differences between pre-therapy and post-therapy were only observed for the number of health investigations in the PPT group which, surprisingly, increased with a large effect size (d = 1.19 95% CI 1.12–2.88, P = 0.03). The same trend towards increased utilisation were observed for every outcome measure in PPT besides outpatient appointments. Conversely, all outcome measures showed an improvement after CBT apart from the number of health investigations which marginally increased.

CBT significantly decreased GP appointments at 6 months follow-up compared with PPT with a large effect size (η2 = 0.5, p < 0.05). A similar trend was seen for total cost (η2 = 0.5, p < 0.06) with each PPT patient costing £790 more on average than their CBT counterparts during the 6 months after therapy.

**Conclusion:**

Whilst CBT appears to be efficacious in the short-term, PPT caused significantly increased healthcare utilisation compared with CBT in the 6 months after therapy. This aligns with similar studies that demonstrate a ‘sleeper effect’ in which patients who receive PPT, but not CBT, deteriorate before improving over long-term follow-up.

Additional research is needed to correlate this data with symptoms and capture the long-term benefits of these psychotherapies for MUS.

